# What is formulation in psychiatry?

**DOI:** 10.1017/S0033291723000016

**Published:** 2023-04

**Authors:** Gareth Owen

**Affiliations:** Department of Psychological Medicine, Institute of Psychiatry, Psychology and Neuroscience, 16 De Crespigny Park, London SE5 9RJ, UK

**Keywords:** Formulation, biopsychosocial model, diagnostic hierarchy, verstehen, philosophy of psychiatry, medical education

## Abstract

The practice of formulation has been both championed and severely criticised within clinical psychiatry and interest in formulation within the teaching of clinical psychiatry is at a low ebb. This article traces the history of the biopsychosocial model, the concept of diagnostic hierarchy and the role of ‘verstehen’ (or intersubjective meaning grasping) in the clinical assessment. All three of these concepts are considered relevant to the practice of formulation. Responding to challenges aimed at these concepts, it argues that formulation in psychiatry needs resuscitating and rethinking and provides some recommendations for a practice of formulation fit for the 21st century.

*When assessing the same patient, two experts may produce two similar summaries, but two different formulations. This is the fundamental difference: a summary is descriptive, whereas a formulation is analytical and evaluative… formulating a case with clarity and precision is probably the most testing yet challenging and crucial part of a psychiatric assessment*

(The Maudsley Handbook of Practical Psychiatry, 2014)

The challenge of bringing understanding of meaning (‘verstehen’) and explanation (causal knowledge) together in an individual case is the problem of *holism* in psychiatry. In practical psychiatry this is understood as *formulating* a case. Doing this seems both necessary (it is not in the interests of a person to be divided into two by psychiatric assessment and left as such) and attractive (most patients want their experiences to be understood whilst also offered interventions that make a difference). Yet formulation is an activity which has been both championed and severely criticised within psychiatry. In recent times psychiatry has moved away from it, let it fade or has delegated it to psychotherapy. In this paper, I will start from the premise that formulation needs resuscitating but also that it requires some rethinking.

Understanding formulation first requires consideration of the ‘biopsychosocial model’.

## The biopsychosocial model

A key figure in the origins of the biopsychosocial model is Adolf Meyer. Meyer was a Swiss psychiatrist who had trained in Kraepelin's school and had also been influenced by Freud. He immigrated to the USA in his mid-20s where from 1910 he energetically set up the Johns Hopkins University psychiatry programme. In the USA, he was influenced by American pragmatic philosophy, received foreign visitors to Johns Hopkins (including the influential British psychiatrist Aubrey Lewis) and was President of the American Psychiatric Association in 1927/8 having a large subsequent influence on US psychiatry and some on British.

**Fig. 1.**
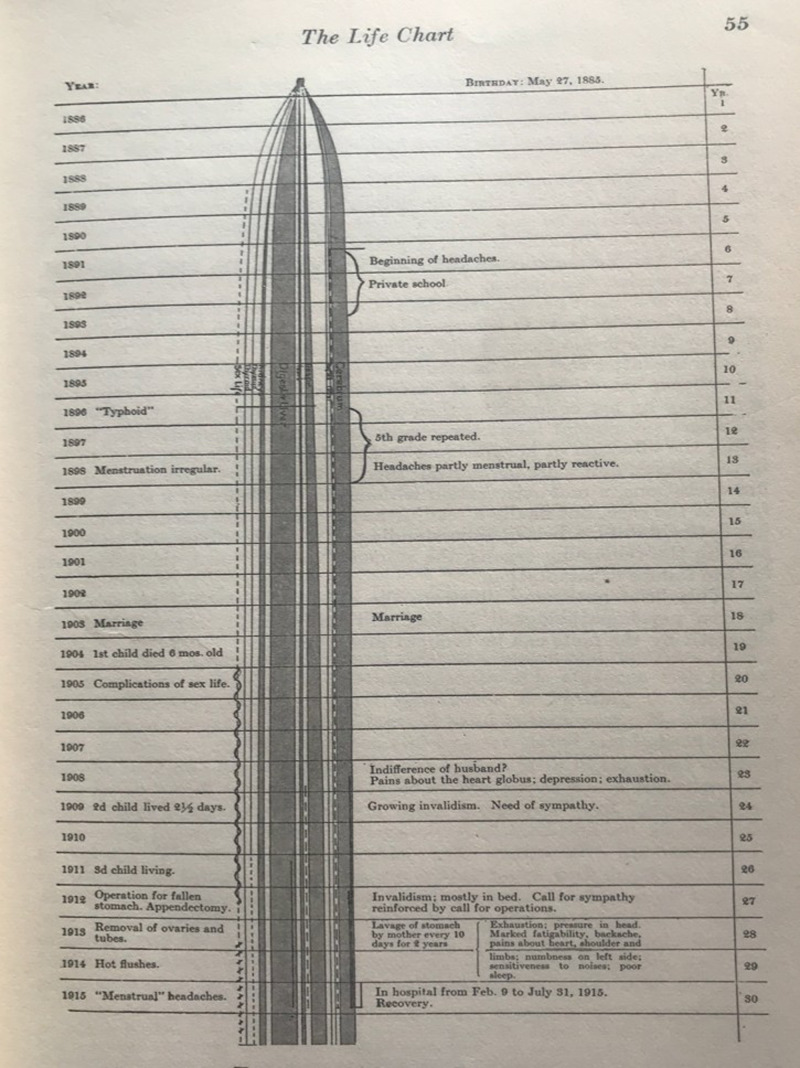
Meyer's example of a life chart (Meyer, 1951, p. 55).

Meyer's central ideas were holism and integration. He thought that the person comes to the psychiatrist via ‘a complaint’ and that the complaint had to be understood in terms of the life course of all their organ systems, their instincts and their life events and formulated as a ‘psychobiological reaction’. So, for example, a person with fatigue, low mood and diffuse somatic complaints required not a diagnosis but a life course analysis and a formulation. A key tool he taught for collecting information was the ‘life chart’ (see Fig. 1).

Meyer became antipathic to diagnosis as a process of identifying a case with a standard entity preferring ‘a formulation of the available facts of each case in terms of ‘an experiment of nature’’ (Meyer, 1951, p. 65). His concept of a ‘fact’ was anything that made a difference to the psychobiological reaction so it could be, for example, an aspect of heredity, an infection, an interruption to instinctual life or a loss of social role.

This was a different approach. It opened up a wide arena of biological, psychological and social causal factors to consider and did not aim to reduce the clinical method to identification of biological causes or allocate cases to clinical entities on a one-to-one basis.

‘The study of the facts in specified cases can readily be a freer clinical method on a frankly pluralistic basis. This is what characterises our direction of work.’ (Meyer, 1951, p. 69)

Another important feature of Meyer's approach was its therapeutic optimism. The approach was action oriented.

‘The question is: What are the dominant facts and what are the points of attack for modification and adjustments?’ (Meyer, 1951, p. 65)

But Meyer did not give much clarity on how to select from the potentially vast amounts of historical data and admitted that the life chart ‘may be unwieldy, and form a ‘long story’’ and have ‘an apparent lack of pointedness’. He acknowledged that ‘it is somewhat difficult to control the time relations and causal interdependence of the events’ (Meyer, 1951, p. 53).

The legacy of Meyer is mainly general. He articulated an early form of the biopsychological model and a pluralistic concept of causation. But he left details unclear and into the vacuum came other theories no less dogmatic than the Kraepelinian nosology Meyer was reacting against. In the context of the USA, this was Freudian doctrine and the concept of formulation was taken forward along those lines, influencing DSM I and II (Ghaemi, 2010).

Another important event in the evolution of the biopsychosocial model was a paper by liaison psychiatrist George Engel in Science in 1977 (Engel, 1977). Engel expanded the model to medicine generally by criticizing the reductionism of the biomedical model used in general medicine. Engel argued that all presentations (e.g. across diabetes, cardiology, etc.) should be approached biopsychosocially. In other words, the biopsychosocial model was the true medical model, not just a model for psychiatry. But again, details were lacking. Like Meyer, the contribution is mainly general: a criticism of mind–body dualism in medicine and unhelpful narrowness of reductionist approaches to causation; plus a moral appeal to holism and ‘the person’ in clinical care.

It is in the light of this quality of expansiveness and lack of detail, that some of the severe criticisms of the biopsychosocial model can be understood. In the influential post war British textbook *Clinical Psychiatry* (Mayer-Gross, Slater, & Roth, 1960) Meyer's approach was considered ‘almost entirely sterile’ (p. 19). A recent leading British textbook has stated that ‘the concept of ‘formulation’ is too muddled for the examination room’ (Johnstone, Cunningham Owens, Lawrie, Sharpe, & Freeman, 2004, p. 237). Ghaemi (2010) has made similar points but adds an interesting critique of the model's scientistic takeover of verstehen which he wants to free from systems theory.

A version of the biopsychosocial model which has a more practical bent and which is not mentioned by Engel in his 1977 paper, or by Ghaemi, is what is sometimes known as the ‘4P model’ (Bolton, 2014). The four Ps stand for different types of causation: predisposing, precipitating, perpetuating and protecting and are applied to three domains: biological, psychological and social (see Table 1). Rational treatment is directed at modifying (if possible) any of the 4Ps (protecting causes being ones to support or enhance).

**Table 1.** The ‘4P model’

Domain	Cause	Treatment
Biological	Predisposing Precipitating Perpetuating Protecting	
Psychological	Predisposing Precipitating Perpetuating Protecting	
Social	Predisposing Precipitating Perpetuating Protecting	

The origins of this model are somewhat obscure but seem to lie in the application of epidemiological thinking to the Meyerian teaching mainly by psychiatrists at the Maudsley in Britain^†^^1^.

A simple case illustrates.

A 40-year-old single male asylum seeker presents to his general practitioner with distress. His mood has been low for 6 months and he sleeps poorly, has lost weight and describes poor concentration. He is drinking vodka daily. He has described torture for political activity. He is supported financially by friends in London and has been unable to work since arriving 1 year ago. The general practitioner refers to a psychiatrist.

Table 2 shows how relevant P's can be picked out across domains and treatment pointed to modify them. Note some causative factors are not modifiable and highlighting areas of powerlessness may be one of the strengths of the model.

**Table 2.** Application of the 4P model

Domain	Cause	Treatment
Biological	Sleep deprivation Alcohol	Sleep advice, short-term hypnotic medication Alcohol education and reduction
Psychological	Stress/anxiety	Psychoeducation on anxiety Anxiety management techniques
Social	Migration Immigration status uncertainties Social and cultural isolation	– Social and legal advocacy Support groups

The key feature here is evidence-based causation. Unlike the unwieldy Meyerian collection of free facts, each P (from the 4Ps) identified in the assessment of the case needs to be one with an evidence base as to its causative potential (rather than a guess as to its causative potential) and each treatment needs to be one with evidence that the intervention modifies the cause. This disciplines the selection of ‘facts’ and makes the treatment pointed.

The 4P model has fallen out of use – eclipsed by DSM-III and treatment algorithms based on its categories. It suffers from the problem that causative effects are often unknown, or when known multiple and small and that they apply to groups rather than individuals. That can make it challenging to apply.

Another version of the biopsychosocial model originating in DSM-III needs some mention – the multi-axial system. This was a sort of bolt on to the neo-Kraepelinism of DSM-III (Williams, 1985). It is an attempt to provide a biopsychosocial assessment by specifying each case in terms of five axes. DSM-III specified the axes as follows:

Axis I – clinical syndrome (e.g. schizophrenia, major depression)

Axis II – lifelong disorders or handicaps (personality disorder or mental retardation)

Axis III – associated physical health problems

Axis IV – severity of psychosocial stressors

Axis V – highest level of social and occupational functioning in last year

The intention was to give a more holistic picture of a case by placing a psychiatric diagnosis (axis 1) within a wider context of personality, learning abilities, physical health and psychosocial stresses. The multi-axial system like the 4P model has rather died out and a little remarked upon change in DSM-5 is that the multi-axial system has been removed^2^.

**Fig. 2.**
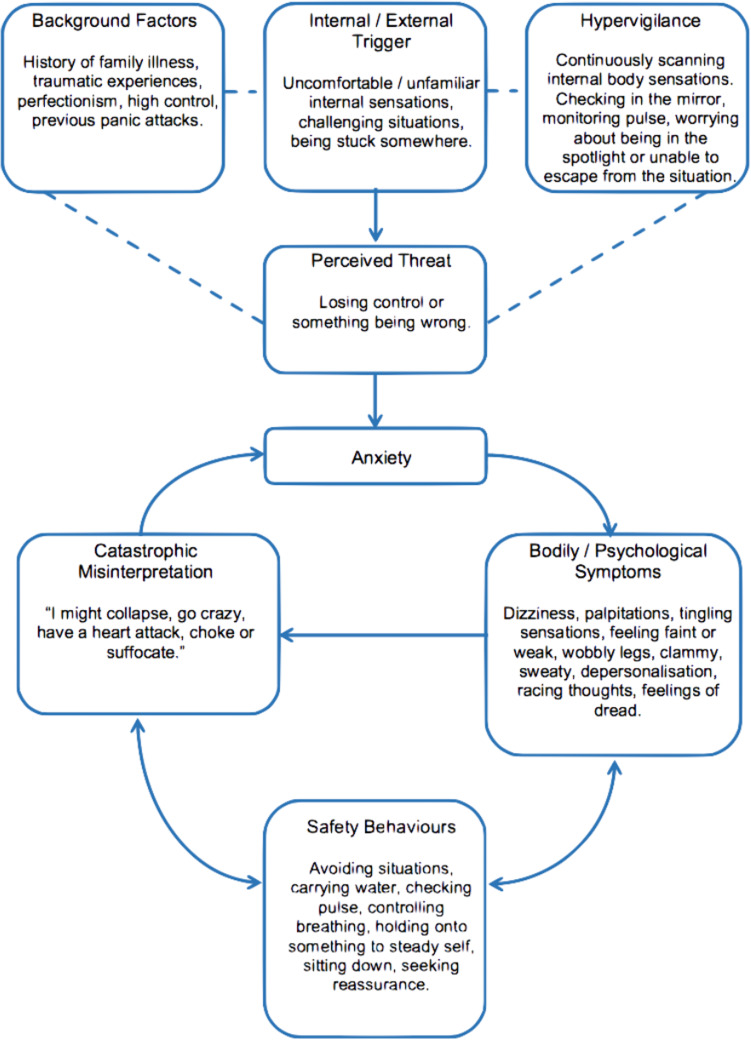
A cognitive and behavioural formulation of anxiety. *Source*: thinkcbt https://thinkcbt.com/cognitive-behavioural-therapy

So where are we now with formulation? The active areas are in psychotherapy. Cognitive behavioural therapy makes active use of formulations. Figure 2 gives a typical one for anxiety as a complaint or symptom. Here box and arrow diagrams are constructed to give a mechanistic picture of the 4Ps operating in the psychological domain using cognitive and behavioural ideas and experimental evidence.

Psychoanalytic psychotherapy also speaks of formulating a case. Within this framework, personal/developmental history is linked to ideas about unconscious dynamic mechanisms and conflicts which are grasped interpersonally through the experience of transference. The formulation is typically given in prose text sometimes with a 4P structure.

So formulation has come to be something primarily psychological in the sense of done by psychotherapists. But that leaves gaps. The diagnostic concept is left out. The biological and social domains are left out (both domains have a 4P structure). In leaving these out the holism or integrative intent of the biopsychosocial model is not achieved. This was not the idea of formulation in Meyer and others.

Currently, formulation is not in a good place. It has either been displaced from teaching and research or reduced to a psychological domain. Some of those developments are understandable – the biopsychosocial model has, after all, not delivered on its promises. But the requirement for some degree of holism and integration is not going to go away. So, a re-think on formulation is needed. In what follows I will try to make a contribution under three headings: (1) the causal nexus, (2) the diagnostic hierarchy and (3) verstehen psychiatry.

## The causal nexus: the connection between phenomena

The biopsychosocial model is a sort of hybrid of anti-reductionistic philosophical complaints and attention to the causal nexus across plural domains (biological, psychological, social).

Key characteristics of causation are: intervention, prediction and subsuming under a *general* law (‘nomological’). Here the 4P model offers an advance on the vagueness of Meyer and Engel and brings the biopsychosocial model into focus as a causal model answering to the demands of empirical science. Further advances on Meyer and Engel are also worth noting. Firstly, there are recent contributions in the philosophy of science (Cartwright, 1983; Kendler & Campbell, 2009; Pearl, 2000; Woodward, 2003) which displace physics as the prototypical model of causation – especially in complex systems. James Woodward's non-reductive, interventionist theory of causation can be read as a more worked out version of Meyer's dictum that the question is always ‘what are the points of attack for modification and adjustments’. A casual relationship is a relationship exploitable for purposes of manipulation and control according to Woodward (2008, p. 137). It is about ‘making things happen’. So, for example, if it is possible to intervene on social variables like ‘poverty’ (e.g. as a public health intervention) in ways which show a stable relationship with improving anxiety (and without excessive side effects elsewhere) then poverty would be as *bone fide* cause of anxiety as physical variables such as a polygenetic risk score. Furthermore, a mix of variables across domains (e.g. poverty and alcohol) would be a superior cause if it were to have a more stable relationship with anxiety than one variable alone especially if the mix affords opportunities for intervention. This is a pluralistic theory of causation with an emphasis on making things happen which would have delighted Meyer. Recent detailed examples of this causal version of the biopsychosocial model can be found in Kendler's model of predictors of depression (Kendler & Prescott, 2006). Bolton and Gillet, taking inspiration from Engel, have also discussed it in relation to big data clinical informatics and the potential for integrated mind/body healthcare (Bolton & Gillett, 2019).

But how should we apply general statistical models to the individual case? Kendler's model of depression applies to predicting depression in groups. A randomised controlled trial result for an anti-depressant specifies means not individuals. But formulation is ‘idiographic’ – it is about the case of *N* = 1.

The hardness of this question is easier to see from the perspective of law than from the perspective of medicine. Imagine a judge having to decide if Kendler's model of depression can be used for the case in front of them in relation to say a court claim regarding the cause of depression. Is social stress a causative factor in *this* person's claim? What is to say that *this* individual is not similar to ones in the original dataset which actually weakened the causal relationship rather than made it? The judge must decide about *this* case not the general case. In law the problem of how a judge should use statistical data is called the ‘G2i’ or the general to individual problem (Faigman, Monahan, & Slobogin, 2014). Note, the G2i problem concerns the logic of single case judgement rather than questions about the generalisability or representativeness of a statistical study. It is also not about verstehen.

There are techniques regarding single case judgement and I suggest they could have more attention in research and teaching to make the 4P model more applicable to formulation. These include checking that the statistical cause (whether biological, psychological or social) is actually present in the individual case, understanding the statistical strength of an effect (focusing on large ones), looking for evidence of concomitant variation (in other words, in this individual when the factor changes, does the presenting complaint change?). These techniques will reduce the chances that applying the 4P model will be merely general and unresponsive to the G2i problem.

## The diagnostic hierarchy

The multi-axial system of DSM-III with its distinction between axis I and axis II disorders acknowledged a difference of kinds within the concept of mental disorder. DSM-5 backtracks on that and flattens the diagnostic hierarchy.

The diagnostic hierarchy is a response to the excesses of ‘unitary psychosis’ (Berrios & Beer, 1994) or the view that there is essentially only one kind of mental disorder and that this is on continuum with health. Diagnostic hierarchy has its origins in the traditions of clinical method, especially Jean-Pierre Falret (known for observations of what we now call bipolar), Karl Ludwig Kahlbaum (known for his observations of catatonia) and Emil Kraepelin (famous for his nosological system). Importantly, it addresses all mental disorders and does not exclude, as some discussions have done (Foulds & Bedford, 1975), organic mental disorders. Furthermore, the concept is not essentially about symptoms but rather about clinical kinds and broad relations between them. The hierarchical nature of diagnostic kinds and their laws of relation was distilled out from the clinical literature by Karl Jaspers in his *General Psychopathology* (Jaspers, 1962) and Kurt Schneider in his *Clinical Psychopathology* (Schneider, 1959). The model is holistic and integrative in that it looks to discern broad groupings of mental disorder and assist the diagnostician in a general approach. The model, in contrast to the over 300 diagnoses and criterion sets in DSM-5, is extraordinarily simple (see Table 3).

**Table 3.** The diagnostic hierarchy (adapted from Jaspers/Schneider)

**Group I** Physical illnesses with psychological consequences (Organic psychiatry – diseases)
**Group II** Schizophrenia and major affective disorder (Qualitative shifts from health)
**Group III** Common mental illnesses, personality, neurodevelopmental variations, reactions to stress (Variations of human nature)

Jaspers wrote that ‘The three main groups of disorders are essentially different from each other’ (Jaspers, 1962, p. 610). With group I it was possible to speak of diseases – as in Alzheimer's neuropathology – but this did not mean that group I diagnoses had no mental structure or phenomenology. In fact, Jaspers makes use of much richer phenomenological accounts of, for example, delirium than is found in DSM-5 or in contemporary medical or neurological texts which focus on level of consciousness, attention and level of motor activity. Complex psychotic experiences can arise in delirium with all the features of a psychiatric disorder. They are organic (group I) – not group II – and the term ‘symptomatic psychosis’ was used.

With group II, it was possible to speak of what is health and what is not in phenomenological terms, i.e. qualitative shifts in mental state. Jaspers and Schneider were reticent about using the concept of disease here. Hence, clinical phenomenology became an important way to explore the boundaries of this group. With group III, the phenomena merged with everyday life and normal human variations. It is important to note that this did not mean that group III disorders could not be severe or disabling. Jaspers' early case studies on psychosis are all studies of complex *reactive* psychoses (Jaspers, 2007) – all severe and disabling but, on closer analyses, merging into normal human variation and reactions to life events (i.e. they were group III).

The laws of relation between the groups are that group I can include the members of group II and III and group II can include the members of group III. The diagnostic convention is to formulate on the basis of the diagnosis closest to group I (organic) if there is evidence to support it. So, for example, anxiety and personality disturbance (phenomena occurring in group III) can be included in a group I or II diagnosis because they can arise there too. Passivity symptoms, primary delusions, flight of ideas and pervasive melancholic symptoms (phenomena occurring in group II) do not arise in group III but can in group I so, similarly, the rule is to diagnose a group II disorder only if there was evidence for the absence of a group I disorder that could manifest them. Disorientation, confabulation and seizure (phenomena occurring in group I) do not arise in either group II or III so are directly indicative of a group I disorder.

Multiplication of diagnoses is constrained by the relations in this hierarchy. In the collection and interpretation of clinical information, one is always asking ‘is the phenomena accountable to group I or II or III?’ For Jaspers and Schneider, assessment also demands a grasp of the full range of each group [as in the examples above of the symptomatic psychosis (organic) and the reactive psychosis (human variation)].

None of this precludes blurring and messiness and there may be more than one group operating in any one case. But that is another matter: complexity and uncertainty is endemic across all clinical work. These groupings are meant as ‘ideal types’ or family resemblances.

More recently, psychiatrist Paul McHugh at Johns Hopkins proposed another version of a diagnostic hierarchy giving it some ontological underpinnings. He proposes four emergent features (or sets) of mind/brain that form distinct ‘intelligible’ levels (McHugh, 2012):

1. the ‘intrinsic’ set that includes features such as consciousness itself and other staples of the mind such as perception, memory, language and affect.
2. The ‘self-differentiating’ set that includes each individual's characteristic intelligence (IQ), temperament and maturational stage.
3. The ‘teleological’ set that encompasses those features of mind that organise and inform goal-directed behaviours such as appetites, drives, intentions, choices and habit conditioning.
4. The ‘extrinsic/experiential’ set comprised of the features that, responding to life events, social networks, education and all the influences of family life, occupation and culture, bring about individuation, psychosocial development and character formation.

This hierarchy of levels is linked to the approach McHugh and Phillip Slavney use (McHugh & Slavney, 1998) to teach psychiatrists a pluralistic approach to psychiatric assessment. McHugh relates the four ‘intelligible’ levels to diagnosis. Diseases (and here, *unlike*, Jaspers/Schneider, McHugh groups dementia, delirium with schizophrenia and bipolar) are rendered ‘intelligible’ in relation to level 1. Learning disabilities and personality disorders are rendered ‘intelligible’ in relation to level 2 which is considered inherently dimensional. Eating disorders and addictions are rendered ‘intelligible’ in relation to level 3. Post-traumatic stress disorder and adjustment disorders are rendered ‘intelligible’ in relation to level 4.

An interesting point McHugh makes is that the process of diagnosis established through the clinical method is about ‘intelligibility first, explanation second’ (McHugh, 2012). Indeed, intelligibility is prior to causal explanation: we need to have some prior grasp of *what* kinds of things are being causally explained. We also know from clinical experience that causal knowledge and diagnosis can dissociate. A diagnosis can be intelligible without causal knowledge (e.g. a delirium without a cause found does not stop it being a delirium). This sheds a new light on the 4P model. The 4P model cannot entirely operate without an intelligible diagnostic scheme and can be insufficient for clinical practice due to the fact that causal knowledge can be difficult to come by^3^: we need diagnostic concepts rooted in the clinical method.

I suggest formulation needs to involve more research into, and teaching on, the diagnostic hierarchy. Ontological levels (or what McHugh calls ‘intelligibility’) need to be discussed and expert clinical practices on how levels navigated better understood. DSM-5 has, paradoxically, created an undifferentiating, dessert-like ontology in this regard.

## Verstehen psychiatry

Jaspers introduced verstehen to psychopathology, contrasting it with causal explanation and emphasizing some of its methodological characteristics such as empathy and meaningful connection (Jaspers, 1962). Jaspers is not always clear whether he is giving a broad or narrow definition of verstehen and seems to settle on a narrow one calling it, somewhat confusingly to a contemporary reader, ‘genetic causation’ and implies a type of causation that is different to that known by experimental science. But this other type of causation is never made very clear. Critics of verstehen have come from opposing philosophical camps in psychiatry (positivists and anti-positivists), so verstehen has been a little bit like an approach stuck in the middle of a dual carriageway at risk of impact from oncoming traffic in both directions. This manifested in the DSM-III drive to improve inter-rater agreement or reduce ‘noise’ in psychiatric diagnosis (verstehen always attracted suspicion from positivists as a source of noise because of its resistance to operationalisation and its apparent subjective character). Verstehen has also attracted suspicion from anti-positivists as power oppression of lived experience by psychiatric orthodoxy because it presupposes that a form of second-personal understanding of another is possible. If there is no human nature (a theme of anti-positivist, poststructuralist philosophy) then we can only presuppose subjective experience.

To my view, the simultaneous critique of verstehen as incompatible with inter-subjectivity *because* it cannot be operationalised for inter-rater reliability AND *because* it wrongly presupposes the very possibility of inter-subjectivity amounts to a reductio ad absurdum of the critique as a whole. I think verstehen survives the critique and continuing to think it through for formulation is necessary.

Verstehen can be given a better definition than ‘genetic causation’ and there are better and worse ways of doing it which make it accountable to constructive criticism. Verstehen is a core part of clinical phenomenology (Broome, Harland, Owen, & Stringaris, 2012) and is also important to grounding judgements in psychiatry about freedom^4^.

There is a further way in which verstehen captures a core concept in formulation. This is the idea of *reaction* articulated by Meyer but cluttered up by his somewhat unclear psychobiological thinking.

Reaction is a fundamentally expressive phenomenon and thus in the remit of verstehen. Ernst Kretschmer understood this with his work on character and ‘key experiences’ or experience which can elicit the most characteristic reaction from a given personality that can bring them into dysfunction. He wrote:

A personality reaction… represents *the purest and most significant expression of the individuality as a whole*; it is entirely specific, limited by characterological make-up and governed by circumstance. It only arises when a certain definite appropriate experience influences a certain definite individual.

The experiences which are especially calculated to elicit the most characteristic responses from a given personality are termed ‘*key experiences*’. Character and key experiences fit together like a lock and key… especially if certain environmental factors contribute to the combination. (Kretschmer, 1952, p. 252).

The expressive nature ultimately has to be grasped, as it were, *in vivo*, in the particularities and experience of the individual's unfolding in time and shown as such (e.g. in biographical case studies and in descriptions of ways of experiencing). Some of the best examples of this kind of understanding come from literature. Protypes are Shakespeare's characters of Hamlet and Ophelia. When Hamlet hears news of the murder of his father he famously expresses ‘The time is out of joint: O cursed spite, That even I was born to set it right’ (Shakespeare, 1987, pp. 1.5, 188). Both Hamlet and Ophelia go mad in reaction to ‘key experiences’ and Shakespeare shows us how without making use of a single experiment.

Whole units of experience and action which may be extremely *complex* in terms of 4P causal analysis, or not diagnostically precise, may be phenomenologically *simple* nonetheless. But we experience them in time, or in the flow of a life process. We cannot repeat, or causally manipulate them. Rather, we share and show them in individual cases. Doing this can be a core component of what it is to formulate a case because it renders something complex simpler and gives a focus and direction to care and treatment.

I suggest broadening the definition of verstehen psychiatry, allowing it to signify an interpretative process of grasping human expressivity and intentionality. It captures key aspects of phenomenological psychiatry, methods of understanding *v.* explanation, ways to understand freedom/unfreedom of action as well as concepts like ‘reaction’ outlined here.

## What is formulation?

In summary, formulation is a synthetic step in the psychiatric assessment involving clinical judgement. In formulation, ontological (diagnostic hierarchy), causal (biopsychosocial model) and meaning (verstehen) perspectives combine to give an overall picture of an individual case and a basis for treatment and care. It involves a stratified diagnostic scheme to give intelligibility to clinical information, a pluralistic, biopsychosocial 4P mode of causal analysis focused on intervention (and recovery and prognosis) and a verstehen mode of grasping expressivity and intentionality in a human person.

In any formulation, all three approaches will have relevance but the relative emphasis will be guided by diagnostic level. In a group I case, diagnostic precision is more likely, the 4P model will often be simpler and shifted to biology and verstehen, though relevant, more constrained. In a group III case, the fuller 4P and verstehen modes will become more salient and diagnostic precision less likely.

Formulation spans organic psychiatry to the psychiatry of everyday life and is not limited to subspecialities with psychotherapeutic interests. I make three recommendations:

1. Interpret the biopsychosocial model as a 4P causal model and focus on teaching psychiatrists modern pluralistic concepts of causation and techniques for the clinical judgement of causation in individual cases.
2. Understand the diagnostic hierarchy as a guide to clarifying the right ontological level for a formulation. Teaching needs to separate this from DSM criteria sets or aetiology and connect it to the practice of ‘differential diagnosis’ across medicine.
3. View verstehen psychiatry as a cluster of approaches to clarifying the phenomena of meaning and expressivity in clinical presentations. This involves teaching phenomenological psychopathology, methods of understanding *v.* explanation, ways to understand freedom/unfreedom of action as well as concepts like ‘reaction’.

Formulation needs to be taught again. To make progress with these three recommendations, it also needs to be researched as a core topic in academic psychiatry.

## Notes

1. David Goldberg, for example, who ran the Institute of Psychiatry, London in the 1990s taught it. For example, D.P. Goldberg, L. Gask and R. K. Morriss. (2014) Psychiatry in Medical Practice (3^rd^ Edition), Routledge (p. 70).

2. DSM-5 introduced, in its section on emerging measures and models, a cultural formulation interview but this does not impact on the diagnostic structure of the manual. WHO advocates the International Classification of Functioning, Disability and Health (ICF) which has found clinical use in rehabilitation psychiatry but the ICF is not integrated into ICD-11 in the way the multi-axial system was integrated into DSM-III.

3. Even Meyer uses a version of the diagnostic hierarchy *prior to* his psychobiological formulations (‘It has been found to be advisable to *start from* three large [diagnostic] groups’ (italics added). ‘Notes of Clinics in Psychopathology’, in *Collected Papers of Adolf Meyer*. Johns Hopkins Press (1951), p. 144).

4. Verstehen is sometimes assumed to relate to human freedom in the sense of being identified with it. This has meant that verstehen psychiatry has sometimes been an ‘anti-psychiatry’ that asserts a sort of unconditional human freedom against psychiatry. But this is misleading as Ludwig Binswanger, an original proponent of verstehen psychiatry, knew. In his long existential analysis of ‘The case of Ilse’ he uses the methods of verstehen to show how an action of Ilse's which is meaningful is also *compelled* and thus unfree (‘Insanity as Life-Historical Phenomenon and as Mental Disease: the Case of Ilse’. In, *Existence*, Edited by May, R. et al., p. 234). Part of the value of verstehen for psychiatry is to help differentiate the freedom or unfreedom of actions (this is relevant to assessment of mental capacity or competence).

## Footnotes

† The notes appear after the main text.
